# Identification and validation of a lactate metabolism-related six-gene prognostic signature in intrahepatic cholangiocarcinoma

**DOI:** 10.1007/s00432-024-05723-4

**Published:** 2024-04-16

**Authors:** Chen Sang, Li Yan, Jian Lin, Youpei Lin, Qiang Gao, Xia Shen

**Affiliations:** 1 Department of Liver Surgery and Transplantation, Liver Cancer Institute, Zhongshan Hospital, and Key Laboratory of Carcinogenesis and Cancer Invasion (Ministry of Education), Fudan University, Shanghai, China; 2 Department of Hematology, Renmin Hospital of Wuhan University, Wuhan, China; 3 Jinshan Hospital Center for Tumor Diagnosis & Therapy, Jinshan Hospital, Fudan University, Shanghai, China

**Keywords:** Intrahepatic cholangiocarcinoma, Lactate metabolism, Prognostic model, Immune microenvironment

## Abstract

**Purpose:**

Intrahepatic cholangiocarcinoma (iCCA) is a highly malignant and fatal liver tumor with increasing incidence worldwide. Lactate metabolism has been recently reported as a crucial contributor to tumor progression and immune regulation in the tumor microenvironment. However, it remains poorly identified about the biological functions of lactate metabolism in iCCA, which hinders the development of prognostic tools and therapeutic interventions.

**Methods:**

The univariate Cox regression analysis and Boruta algorithm were utilized to identify key lactate metabolism-related genes (LMRGs), and a prognostic signature was constructed based on LMRG scores. Genomic variations and immune cell infiltration were evaluated in the high and low LMRG score groups. Finally, the biological functions of key LMRGs were verified with in vitro and in vivo experiments.

**Results:**

Patients in the high LMRG score group exhibit a poor prognosis compared to those in the low LMRG score group, with a high frequency of *TP53* and *KRAS* mutations. Moreover, the infiltration and function of NK cells were compromised in the high LMRG score group, consistent with the results from two independent single-cell RNA sequencing datasets and immunohistochemistry of tissue microarrays. Experimental data revealed that lactate dehydrogenase A (*LDHA*) knockdown inhibited proliferation and migration in iCCA cell lines and tumor growth in immunocompetent mice.

**Conclusion:**

Our study revealed the biological roles of *LDHA* in iCCA and developed a reliable lactate metabolism-related prognostic signature for iCCA, offering promising therapeutic targets for iCCA in the clinic.

**Supplementary Information:**

The online version contains supplementary material available at 10.1007/s00432-024-05723-4.

## Introduction

Cholangiocarcinoma (CCA) is broadly categorized into intrahepatic CCA (iCCA), perihilar CCA (pCCA), or distal CCA (dCCA) based on its anatomic origin (Brindley et al. 2021). Recent data have reported an increase in the incidence and mortality of iCCA in Western and Eastern countries (Kelley et al. 2020). Moreover, advanced iCCA has a median overall survival (OS) of < 12 months (Tsimafeyeu and Temper 2021). Therefore, it is crucial to identify factors influencing the prognosis of iCCA for its effective clinical management. Although the tumor-node-metastasis (TNM) staging manual is widely used to classify iCCA patients, it remains limited in predicting prognosis and selecting treatment options for such patients (Edge and Compton 2010). Specifically, the TNM staging approach fails to reveal the biological heterogeneity of iCCA or fully explain the remarkable variations in prognosis and treatment responses even among patients at the same stage. Moreover, the survival of iCCA patients is impacted by other factors, such as age, performance status, and tumor site (Brindley et al. 2021). Accordingly, it is urgently needed to identify reliable prognostic biomarkers that capture tumor biology for increasing the clinical value of prognostic prediction and uncovering new therapeutic targets for iCCA.

The advent of high-throughput sequencing technology and bioinformatics tools now allows for the identification of potential molecular targets in various diseases (Hong et al. 2020), which is beneficial for the development and application of effective targeted therapies for iCCA. Recent studies have elucidated the proteogenomic subtypes and related biomarkers in cancers and systematically explored the heterogeneous immune microenvironment of iCCA and the crucial role of glycolysis and lactate metabolism in this immune microenvironment (Yang et al. 2023; Lin et al. 2022a). Further analyses of the clinical relevance of lactate metabolism to iCCA have provided new pathogenic mechanisms for iCCA (Dong et al. 2020, 2022; Lin et al. 2022b). Emerging data have underscored the significance of lactate metabolism in shaping the tumor immune microenvironment (TIME) (Certo et al. 2021). Moreover, prior studies showed that the accumulation of lactate in TIME negatively impacted the function of immune cells such as CD4^+^ T cells, CD8^+^ T cells, natural killer (NK) cells, and natural killer T cells, while elevating the activity of regulatory T cells and myeloid-derived suppressor cells (Angelin et al. 2017; Peng et al. 2016), which ultimately compromised anti-tumor immunity. Of note, lactate metabolism-related gene (LMRG) signatures have been utilized in predicting prognosis and evaluating TIME in breast cancer, lung adenocarcinoma, and hepatocellular carcinoma (Wang et al. 2021; Yang et al. 2022, 2023; Li et al. 2021). However, limited studies are available on LMRGs and TIME-related lactate metabolism in iCCA (Thonsri et al. 2017), and the biological function of LMRGs in iCCA remains incompletely explored.

Therefore, it may be an important direction for developing treatment strategies and predicting prognosis for iCCA to investigate the biological functions of LMRGs in iCCA and to link tumor metabolism to the TIME represented by lactate metabolism. Herein, this study analyzed the correlation between lactate metabolism and the tumor microenvironment (TME) in iCCA and evaluated the clinical significance of an LMRG prognostic signature. Our results indicated that the molecular signature constructed based on LMRGs could predict the prognosis of iCCA patients. In addition, this study explored the key biological functions of lactate dehydrogenase A *(LDHA*) in the progression of iCCA through in vitro and in vivo experiments, displaying that *LDHA* might be a novel promising therapeutic target for iCCA patients.

## Materials and methods

### Data collection and pre-processing

This study involved the bulk RNA sequencing (RNA-seq) and whole-exome sequencing (WES) data of 255 iCCA patients in prior studies (Lin et al. 2022b; Dong et al. 2022). In addition, a validation dataset was collected from a previous study (Deng et al. 2023), and single-cell RNA sequencing (scRNA-seq) datasets were obtained from published literature (Ma et al. 2021; Song et al. 2022).

### Differentially expressed genes (DEGs) in the high and low LMRG score groups

DEGs in the high and low LMRG score groups were screened and analyzed using DESeq2 (Love et al. 2014), with |logFC|> 1 and adjusted false-discovery rates < 0.05 as the screening criteria. Then, partial least squares-discriminant analysis (PLS-DA) was performed to determine the profile differences between the high and low LMRG score groups. Subsequently, the R packages ggplot2 and ComplexHeatmap were utilized to generate various plots for the DEGs, such as volcano plots, heat maps, and PLS-DA maps.

### Construction of a lactate metabolism-related prognostic scoring model for iCCA

Two hundred and fifty five iCCA samples with survival information were obtained for the construction of the prognosis risk model, and 84 samples with survival information were used for external validation (Deng et al. 2023). LMRGs associated with the OS of iCCA patients were identified with the univariate Cox analysis, and only LMRGs with *P* < 0.05 were selected for subsequent analysis. The Boruta algorithm, an all-relevant feature selection wrapper algorithm, was utilized to avoid the overfitting of the prognostic model and narrow the range of genes predicting OS (Kursa 2010). LMRGs filtered with the Boruta algorithm were assessed with the multivariate Cox regression analysis. The LMRG prognostic scoring model was constructed based on the expression of each gene and its corresponding importance. The patients were categorized into high and low LMRG score groups using the score formula, with the median score as the threshold. The dissimilarities in OS between the two groups were calculated with the Kaplan–Meier analysis and log-rank test. The sensitivity and specificity of the prognostic scoring model were identified with the receiver operating characteristic (ROC) curve and the corresponding area under the ROC curve (AUC).

### Association of the prognostic scoring model and clinicopathological features

Univariate and multivariate Cox regression analyses were carried out to clarify the effect of the risk score on OS and clinicopathological features, followed by the analysis of the correlation between the expression of these LMRGs and several clinicopathological features. In addition, the prediction accuracy of the risk score and the clinicopathologic features was compared with ROC curves.

### Construction of a prognostic nomogram

To calculate the probability of 1- and 3-year OS, a nomogram was constructed based on independent prognostic factors. The performance of the nomogram was assessed with ROC and calibration curves. Meanwhile, decision curve analysis (DCA) was utilized to further measure the net benefit of the nomogram and single clinical features.

### Functional enrichment analyses

To identify the functional characteristics of the high and low LMRG score groups, a differential gene expression analysis was performed, and Gene Ontology and Kyoto Encyclopedia of Genes and Genomes pathway enrichment analyses were conducted with the R package “ClusterProfiler” (Yu et al. 2012). In addition, Gene Set Enrichment Analysis (GSEA) was utilized to analyze differences in pathway activities between the two risk groups.

### Comprehensive analysis of TIME in the two LMRG groups

To identify the immune infiltration features of iCCA samples, their gene expression profiles were imported to the TIME 2.0 website with 1000 permutations (https://cibersortx.stanford.edu/). The obtained results were utilized to compare the fractions of tumor-infiltrating immune cells in the two LMRG groups. Afterward, the association between LMRG scores and immune cells was established with a correlation analysis.

### Immunohistochemistry (IHC)

This experiment was conducted on the Tissue Microarray (TMA) containing corresponding samples of iCCA tissues from Zhongshan Hospital of Fudan University (Dong et al. 2022). All samples were acquired in accordance with the Code of Ethics of the World Medical Association (*Declaration of Helsinki*) after written informed consent had been obtained. The protocols for sample use in this study were ratified by the Ethics Committee of the Zhongshan Hospital of Fudan University. Standard IHC was performed as previously described (Zheng et al. 2020). Two pathologists blindly measured LDHA, CD56, and CD66b expression in the iCCA tissues of the TMA (Detre et al. 1995). Image J software was employed to determine the mean gray value of LDHA and the number of CD56- and CD66b-positive cells for each patient. This experiment was carried out with primary antibodies against LDHA (Proteintech, Wuhan, China, 19,987-1-AP), CD56 (Proteintech, Wuhan, China, 14,255-1-AP), and CD66b (Abcam, Cambridge, UK, 197,678).

### Cell culture and transfection

Human iCCA cell lines, HuCCT1 (a standardized iCCA cell line with *KRAS* and *TP53* mutations) and 880 (a primary iCCA cell line constructed by our group, which has been revealed to have *KRAS* and *TP53* mutations (Dong et al. 2018)) were cultured in a Roswell Park Memorial Institute (RPMI)-1640 medium encompassing 10% fetal bovine serum (FBS; Gibco, Carlsbad, California, USA), 100 units of penicillin, and 100 mg/mL streptomycin in a 37 °C incubator. Negative control (NC), h*LDHA*-KD#1, h*LDHA*-KD#2, m*Ldha*-KD#1, and m*Ldha*-KD#2 were designed and constructed into pLKO.1 vectors by Xitu Bio (Shanghai, China). Subsequently, these vectors were transfected into HUCCT1 and 880 cells with Lipo3000 (Thermo, Waltham, Massachusetts, USA) as per the manufacturer’s manuals. The used sequences were as follows: h*LDHA*-KD#1: GCCTGTGCCATCAGTATCTTA; h*LDHA*-KD#2: CCACCATGATTAAGGGTCTTT; m*Ldha*-KD#1: GTTCCCAGTTAAGTCGTATAA; m*Ldha*-KD#2: CGTGAACATCTTCAAGTTCAT.

### Western blot analysis

Cells were lysed in Radio-Immunoprecipitation Assay buffer (Beyotime, Shanghai, China) encompassing protease inhibitors (Beyotime) and phosphatase inhibitors (Beyotime). Cellular proteins (20 μg) were separated by 10% sodium dodecyl sulfate–polyacrylamide gel electrophoresis gels and incubated with primary antibodies against extracellular signal-regulated kinase 1/2 (ERK1/2; Abmart, Shanghai, China; T40071), phosphorylation (P)-ERK1/2 (Abmart; TA1015), LDHA (Proteintech; 19,987-1-AP), and glyceraldehyde 3-phosphate dehydrogenase (GAPDH) (Proteintech; 60,004-1-Ig).

### Cell proliferation and migration assays

Cell viability was tested with the cell counting kit (CCK)-8 assay (Dojindo, Kumamoto, Japan). Cells were seeded into 96-well plates (2 × 10^3^ cells/well) 24 h after transfection. At each time point, CCK-8 solutions were added to the plates (10 μL/well) for 1.5 h of incubation, and optical density (OD) values were measured immediately at 450 nm. Cell proliferation was also examined as instructed in the manuals of the Cell-Light 5-ethynyl-2-deoxyuridine (EdU) Apollo567 In Vitro Kit (Ruibo, Guangzhou, China). Microphotographs were captured under a fluorescence microscope and analyzed. For the Transwell migration assay, cells were re-suspended in the RPMI-1640 medium. Next, 2 × 10^4^ cells were placed into the apical Transwell chamber, while 800 μL of medium containing 20% FBS was added into the basolateral chamber. After 24 h, cells remaining in the upper chamber were discarded. Cells migrating across the membrane were fixed and stained, followed by counting in three random fields under a microscope.

### Mouse model establishment

Six-week-old female C57BL/6 J mice (the Shanghai Branch of Beijing Vital River, Shanghai, China) were housed under specific pathogen-free conditions. Animal experiments were performed following institutional guidelines and were approved by the Institutional Animal Care and Use Committee of the Shanghai Branch of Beijing Vital River (2017–0014). A spontaneous mouse model of iCCA was established with reference to previous studies (Affo et al. 2021; Lin et al. 2022a). Thereafter, 25 μg of pT3-EF1a-KRASG12D, 10 μg of PX330-CAG-sgp19, and 5 μg of plasmids containing transposon and SB-luc transposase at a ratio were dissolved in 2 mL of 0.9% NaCl solutions. The obtained solutions were injected into the tail vein of mice in a total volume equal to 10% of body weight in 6 s. Then, the state of the mice was observed, and tumors were dissected and collected after 1 month of modeling.

### Primary cell construction

Tumors of the Kp19 iCCA mouse model were dissected with the Miltenyi Mouse Tumor Dissociation Kit and gentleMACS Octo-Dissociator (Miltenyi) as per the manufacturer’s manuals. The tumors were dissociated into single-cell suspensions, washed twice with phosphate-buffered saline (PBS), re-suspended with a primary cell medium (RPMI-1640 medium with 10% FBS, 50 ng/mL epithelial growth factor, 1 × Insulin-Transferrin-Selenium Solution, 10 μM Y-27632, and 5 μM A83-01), and seeded into a collagen-coated cell culture dish. The cultured cells were passaged over 10 times to remove non-tumor cells such as fibroblasts.

### Subcutaneous tumor model

Cells (2 million) were suspended in 150 μL of PBS and injected subcutaneously into C57BL/6 J mice. The tumor volume was measured every 3 days until the tumor volume reached 150 mm^3^. Mice were euthanized, and tumors were dissected when the tumor volume reached around 2000 mm^3^.

### Statistical analysis

Statistical analysis and visualization were performed with version 4.2.2 of R software (https://www.r-project.org). The Mann–Whitney *U* test was utilized for comparisons between the two groups. Kaplan–Meier survival curves were assessed with the log-rank test. In all tests, two-sided *P* < 0.05 represented statistically significant differences.

## Results

### Identification of LMRGs to construct a prognostic signature

**Fig. 1:**
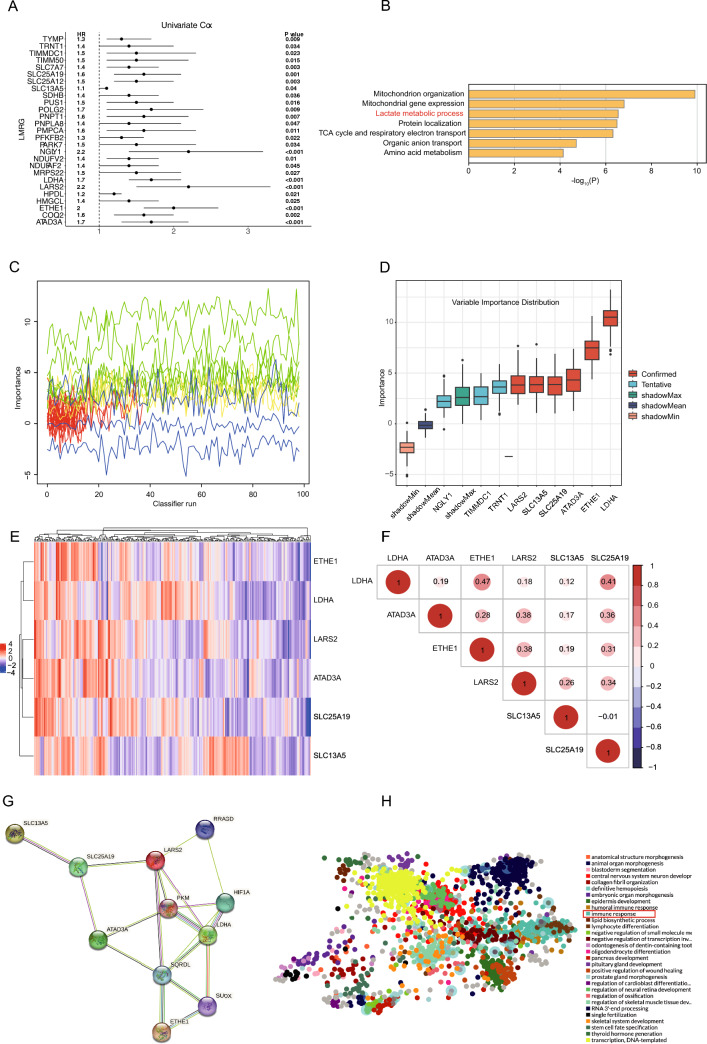
Cox regression and Boruta to filter lactate metabolism-related genes. **A** 27 prognostic LMRGs selected with univariate Cox regression. **B** The GO analysis of the 27 prognostic LMRGs. **C** The selection process of Boruta. **D** The genes selected with Boruta. **E** Expression heat map of the selected 6 LMRGs. **F** Correlation plot for the six LMRGs in iCCA **G** The PPI network of the 6 LMRGs constructed with the STRING website. **H** Transcript factor analysis of the 6 LMRGs with the ChEA3 website

First, the RNA-seq dataset was acquired from a previously published Fudan University (FU)-iCCA cohort (Dong et al. 2022) containing 255 patients. Then, a univariate Cox model was constructed to select prognostic genes among 284 LMRGs (Li et al. 2022), which demonstrated that 27 genes were markedly associated with prognoses (hazard Ratio [HR] > 1, *P* < 0.05) in the FU-iCCA cohort (Fig. 1A). Lactate metabolism-related pathways were obviously enriched (Zhou et al. 2019) (Fig. 1B), highlighting that the selected genes indeed maintain their key lactate metabolism features. Next, a prognostic model was built with the Boruta algorithm (Kursa 2010). In Fig. 1C, the green line corresponds to confirmed features, the red line represents rejected features, and the yellow line denotes features to be identified. The three blue lines represent the importance of minimum, average, and maximum shadow features, respectively.

Eventually, six genes, namely *LDHA*, *ETHE1*, *ATAD3A*, *SLC25A19*, *SLC13A5*, and *LARS2*, were identified and subsequently determined as potential constituents of the LMRG prognostic signature (Fig. 1D). Heat maps were plotted to present the expression of these genes in patients of the cohorts of FU-iCCA and Deng et al. (Deng et al. 2023) (Fig. 1E, Figure S1A). Of note, *LDHA* has been generally considered the central enzyme dictating the lactate metabolism pathway (Thonsri et al. 2017). In our study, *LDHA* was the most significant prognostic LMRG in iCCA (Fig. 1D). The intrinsic correlations among LMRGs were assessed with the correlation analysis and the protein–protein interaction network, which displayed a positive correlation among these six genes (Fig. 1F, G, Figure S1B). Transcript factor analyses with the ChEA3 (Keenan et al. 2019) database indicated that the main functions of co-expressed transcription factors were related to immune responses (Fig. 1H). These results further illustrate that lactate metabolism may be involved in the reprogramming of TIME.

### High and low LMRG scores were tightly associated with the prognosis and mutation profile of patients

**Fig. 2:**
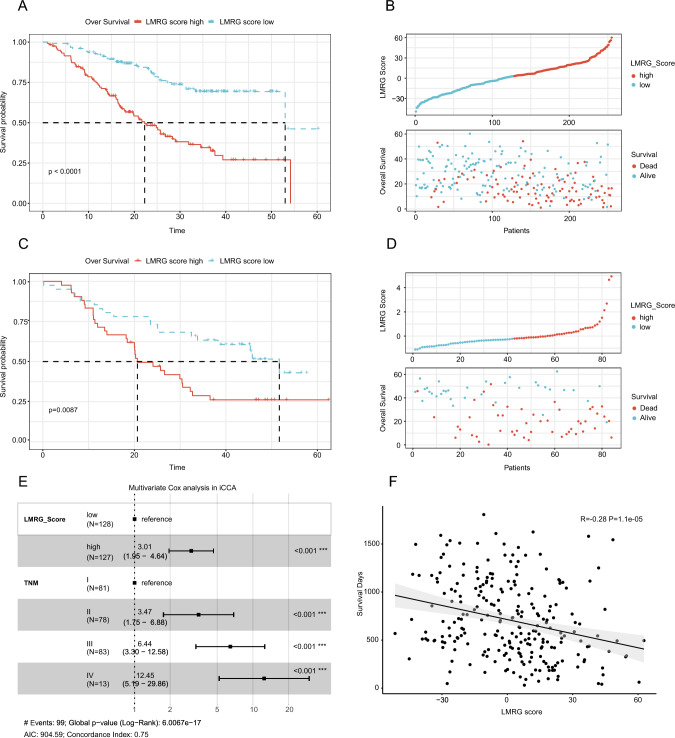
Association of LMRG scores with the prognosis of iCCA. The survival analysis of the high and low LMRG score groups in the cohorts of FU-iCCA (**A**) and Deng et al. (**B**). LMRG score distribution of patients in the cohorts of FU-iCCA (**C**) and Deng et al. (**D**). **E** Multivariate cox regression analysis of LMRG score and TNM stage. **F** The correlation between LMRG scores and survival time

Based on the importance of LMRGs defined with Boruta, the LMRG score was computed with the established formula: LMRG score = (10.17 × *LDHA* expression) + (4.38 × *ATAD3A* expression) + (7.22 × *ETHE1* expression) + (3.86 × *LARS2* expression) + (3.57 × *SLC13A5* expression) + (3.76 × *SLC25A19* expression). Next, patients were allocated into high and low LMRG score groups with the median of the score. As expected, the high LMRG score group showed worse prognoses, which was further validated in the cohorts of FU-iCCA and Deng et al. (Fig. 2A–D). Meanwhile, the HR of the high LMRG score group was 3.01 and 2.57 in these two cohorts according to the multivariate Cox model, respectively (Fig. 2E, Figure S2A), and LMRG scores were negatively correlated with the prognosis of patients (Fig. 2F, Figure S2B).

**Fig. 3:**
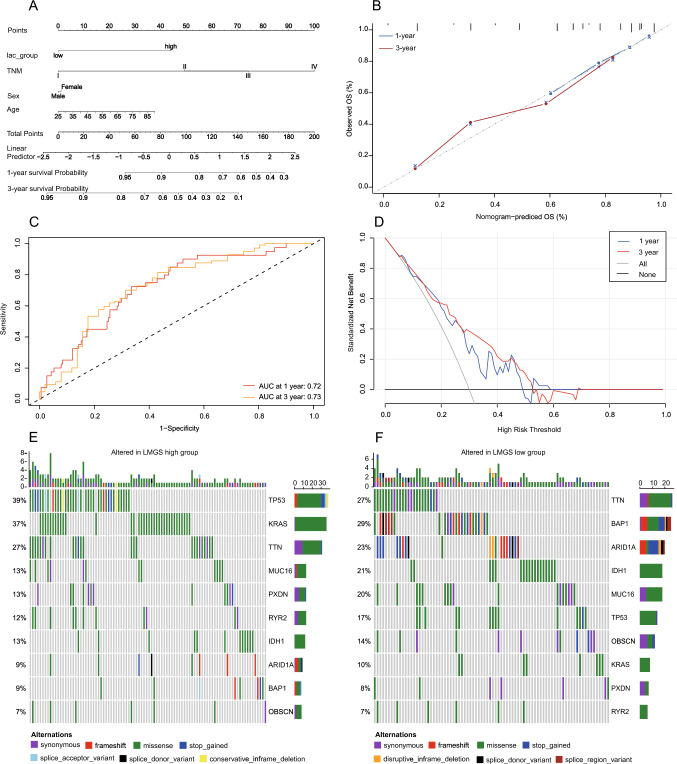
A nomogram and mutation map to estimate the survival rate of iCCA patients. **A** The nomogram for predicting the probability of 1- and 3-year OS. **B** Calibration curves of the nomogram in predicting the probability of 1- and 3-year OS. **C** ROC curves of the nomogram. **D** Decision curves of the nomogram in predicting 1- and 3-year OS. **E** Top 10 mutated genes in the high LMRG score group. **F** Top 10 mutated genes in the low LMRG score group

Clinically, nomograms are frequently utilized to forecast the survival of patients and to calculate points derived from their calculated scores (Balachandran et al. 2015). To predict the 1- and 3-year OS rates of iCCA patients, a nomogram was developed based on independent prognostic markers including LMRG scores and TNM stages to quantitatively assess the prognosis of patients (Fig. 3A, Figure S3A). The calibration curves exhibited the exceptional prediction accuracy of the nomogram in the cohorts of FU-iCCA and Deng et al. (Fig. 3B, Figure S3B). The ROC curves demonstrated the AUC of the nomogram was 0.72, and 0.73 in predicting 1- and 3-year OS, respectively, in the FU-iCCA cohort (Fig. 3C). In the cohort of Deng et al., the AUC of the nomogram was 0.59 and 0.75 in predicting 1- and 3-year OS, respectively (Figure S3C). The clinical validity of the nomogram was assessed with the decision curves. As depicted in Fig. 3D, the nomogram yielded substantial net clinical benefits in both the short and long terms (Fig. 3D, Figure S3D).

To identify genetic variations in the high and low LMRG score groups, the WES data of the FU-iCCA cohort were collected to show gene mutations in these two groups analyzed with the 10 × single-cell technique (Fig. 3E, F). Obviously, *TP53* and *KRAS* mutations were substantially enriched in the high LMRG score group, consistent with our previous finding that both *TP53* and *KRAS* mutations were associated with the poor prognosis of patients (Dong et al. 2022). These results indicate that our LMRG score can effectively characterize the poor prognosis of iCCA patients and has value for further research.

### Prominent impact of the LMRG score on the TIME of iCCA

**Fig. 4:**
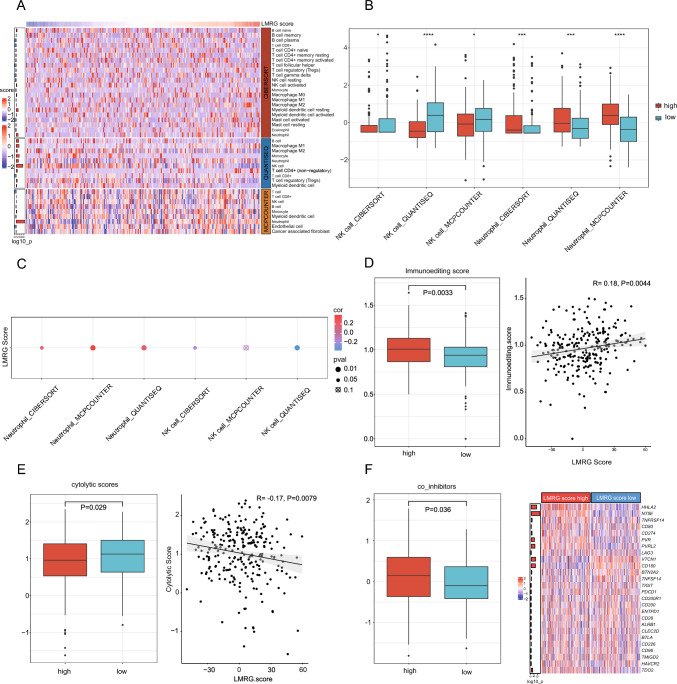
Immune landscape of the high and low LMRG score groups. **A** Heat map of the immune cells evaluated with QUANTISEQ, CIBERSORT, and MCPCOUNTER in the FU-iCCA cohort. **B** The boxplot of NK cells and neutrophils in the FU-iCCA cohort. **C** The correlation of NK cells and neutrophils with LMRG scores in the FU-iCCA cohort. **D** Immunoediting scores in the high and low LMRG score groups in the FU-iCCA cohort. **E** Cytolytic scores in the high and low LMRG score groups in the FU-iCCA cohort. **F** Co-inhibitor scores in the high and low LMRG score groups in the FU-iCCA cohort. *P < 0.05, ***P < 0.001, and ****P < 0.0001

Metabolic pathways, particularly lactate metabolism pathways, play a vital role in remodeling the TIME during the development of numerous cancers (Angelin et al. 2017; Faubert et al. 2017). Thus, the TIME 2.0 website (http://timer.comp-genomics.org) was employed to deconvolve immune cell compositions in the cohorts of FU-iCCA and Deng et al. It was observed that NK cell infiltration was obviously diminished and neutrophil abundance was augmented in the high LMRG score group (Fig. 4A, B; Figure S4A). Furthermore, LMRG scores were also markedly negatively correlated with NK cell infiltration and positively correlated with neutrophil abundance (Fig. 4C, Figure S4B). Then, the immunoediting score was calculated for each tumor, with a score < 1 suggesting the presence of immunoediting. The results manifested that immunoediting scores were prominently higher in the high LMRG score group (Fig. 4D), underscoring that high glycolytic activity mediated by lactate metabolism can potentially suppress cytolytic activity in iCCA. Further results exhibited that cytolytic scores were lowered and co-inhibitor scores were enhanced in the high LMRG score group (Fig. 4E, F), illustrating that active lactate metabolism may contribute to the formation of the immunosuppressive TME in iCCA. LMRG scores also showed negative associations with cytolytic scores and the expression of NK cell functional genes *GNLY* and *GZMM* (Figure S4C, S4D). These results highlight that the immunosuppressive TME may impede NK cell-mediated anti-tumor immunity.

### Validation of LMRG-related immune cell compositions by scRNA-seq analysis

**Fig. 5:**
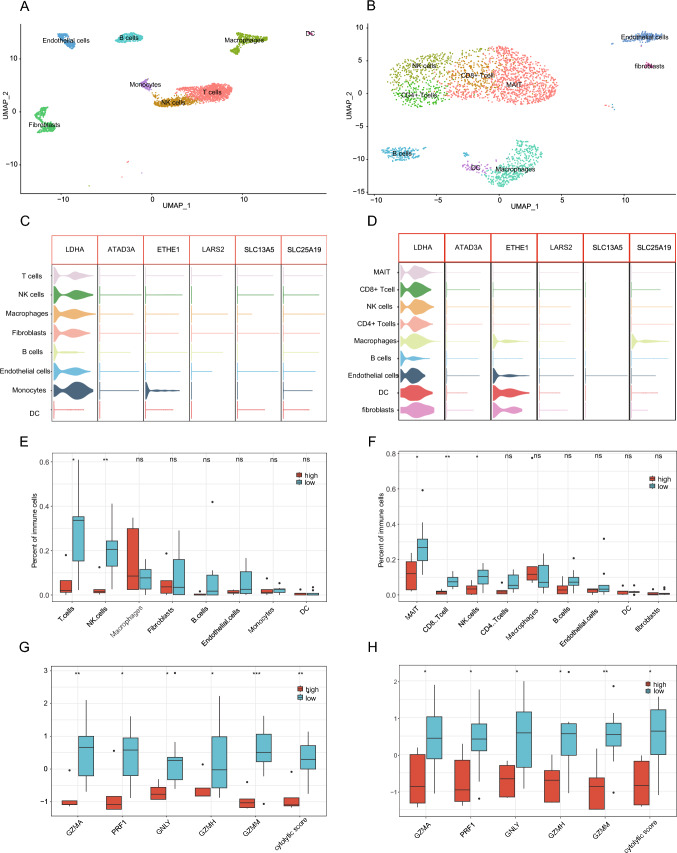
ScRNA-seq of iCCA patients with LMRG scores. The UMAP plot showing the subtypes of immune cells in the cohorts of FU-iCCA (**A**) and Ma et al. (**B**). The violin plot of the six LMRGs in the cohorts of FU-iCCA (**C**) and Ma et al. (**D**). Boxplot displaying the fractions of immune cells in the cohorts of FU-iCCA (**E**) and Ma et al. (**F**) (Wilcox. Test). Boxplot exhibiting cytolytic score-related genes of LMRGs in the cohorts of FU-iCCA (G) and Ma et al. (H). *P < 0.05, **P < 0.01, and ***P < 0.001

To confirm the variations of immune cell compositions between the high and low LMRG score groups, scRNA-seq datasets were obtained from two published references (Ma et al. 2021; Song et al. 2022). Fourteen iCCA patients were selected from both cohorts, respectively, for analysis, and cells were annotated into distinct types shown in UMAP (Fig. 5A, B). The boxplots of the six LMRGs are presented in Fig. 5C and D, which displayed that *LDHA* was expressed at the highest level in the baseline of various immune cells. The expression data of the six LMRGs were extracted and LMRG scores were calculated with the Boruta parameters derived from bulk RNA-seq. The proportions of immune cells in the high and low LMRG score groups are detailed in Fig. 5E and F. Specifically, NK cells markedly enriched in the low LMRG score group, corresponding to a better prognosis. Cytolytic scores were lower and NK cell cytolytic genes were poorly expressed in the high LMRG score group (Fig. 5G, H). All these results validated that lactate metabolism dysregulation may influence TIME by suppressing the infiltration and functions of NK cells. Due to the limitations of prior 10 × single-cell techniques, neutrophils in TIME were not included in this dataset. Intriguingly, prior studies have unveiled that neutrophils are enriched in *KRAS*-mutated iCCA samples, consistent with our results that *KRAS* mutations were obviously enriched in the high LMRG score group (Lin et al. 2022a; Zhang et al. 2023).

### *LDHA* recapitulated the significance of LMRG scores in iCCA

**Fig. 6:**
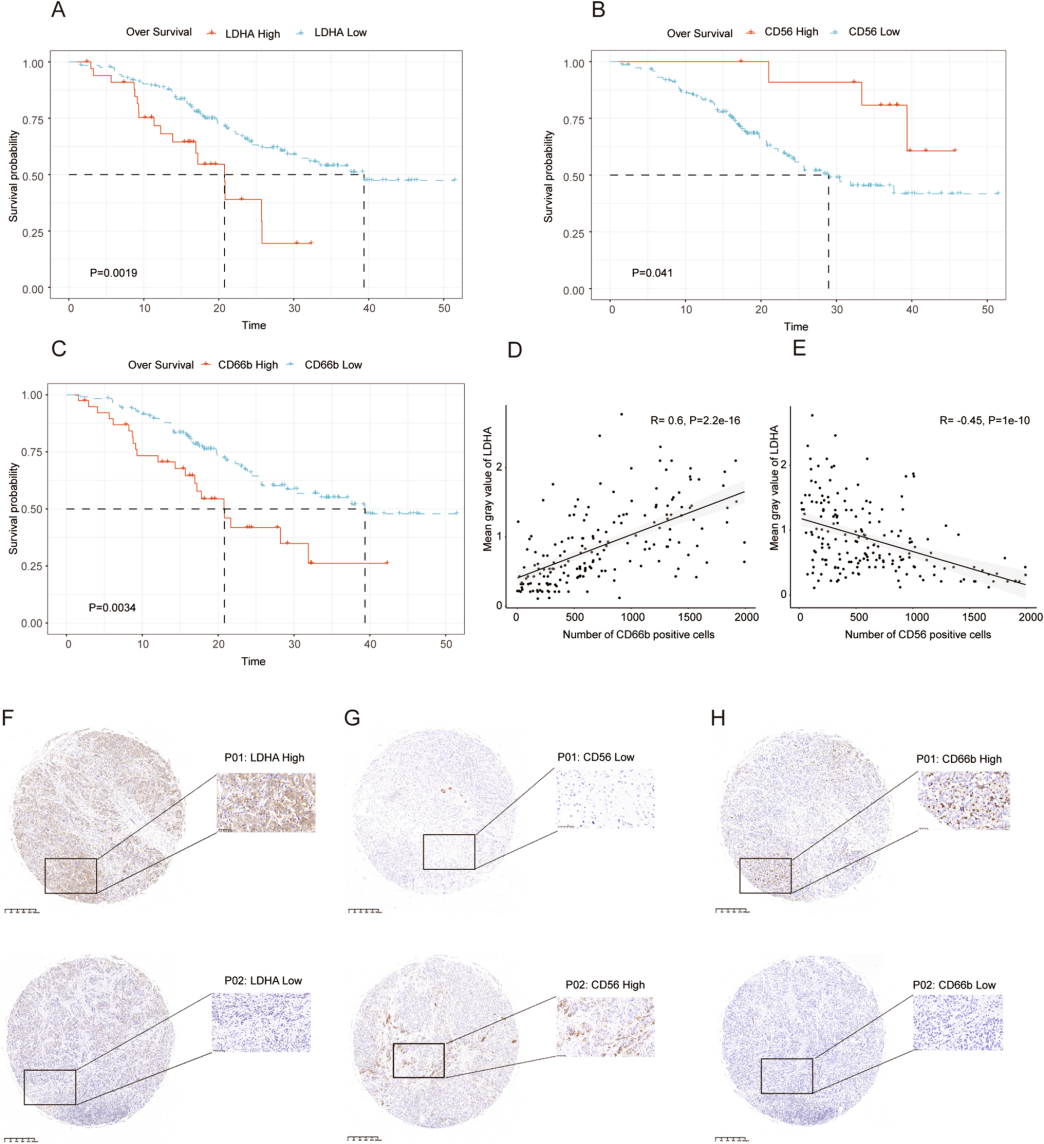
IHC validation of LDHA and CD56. The survival analysis of high and low expression of LDHA (**A**), CD56 (**B**), and CDD66b (**C**). Correlation between LDHA expression and immune cell infiltration (**D**, **E**). Representative samples revealing the expression of LDHA (**F**), CD56 (**G**), and CD66b (**H**) in iCCA patients, respectively

Subsequently, Kaplan–Meier survival curves were plotted to evaluate the prognostic significance of *LDHA*, *CD56* (a gene related to NK cell infiltration), and *CD66b* (a gene related to neutrophil infiltration), respectively. Our results revealed that patients with high *LDHA* and *CD66b* expression exhibited inferior OS outcomes (Fig. 6A, C). In contrast, high *CD56* expression was associated with high OS (Fig. 6B). To further validate our findings in the clinical setting, TMA samples were acquired from the FU-iCCA cohort (Dong et al. 2022). IHC was performed to examine LDHA, CD56, and CD66b expression in iCCA, which demonstrated a positive correlation between LDHA expression and the number of CD66b-positive cells, as well as a negative correlation between LDHA expression and the number of CD56-positive cells (Fig. 6D and Fig. 6E). The representative image is displayed in Fig. 6F–H.

### *LDHA* knockdown suppressed tumor growth in vitro and in vivo

**Fig. 7:**
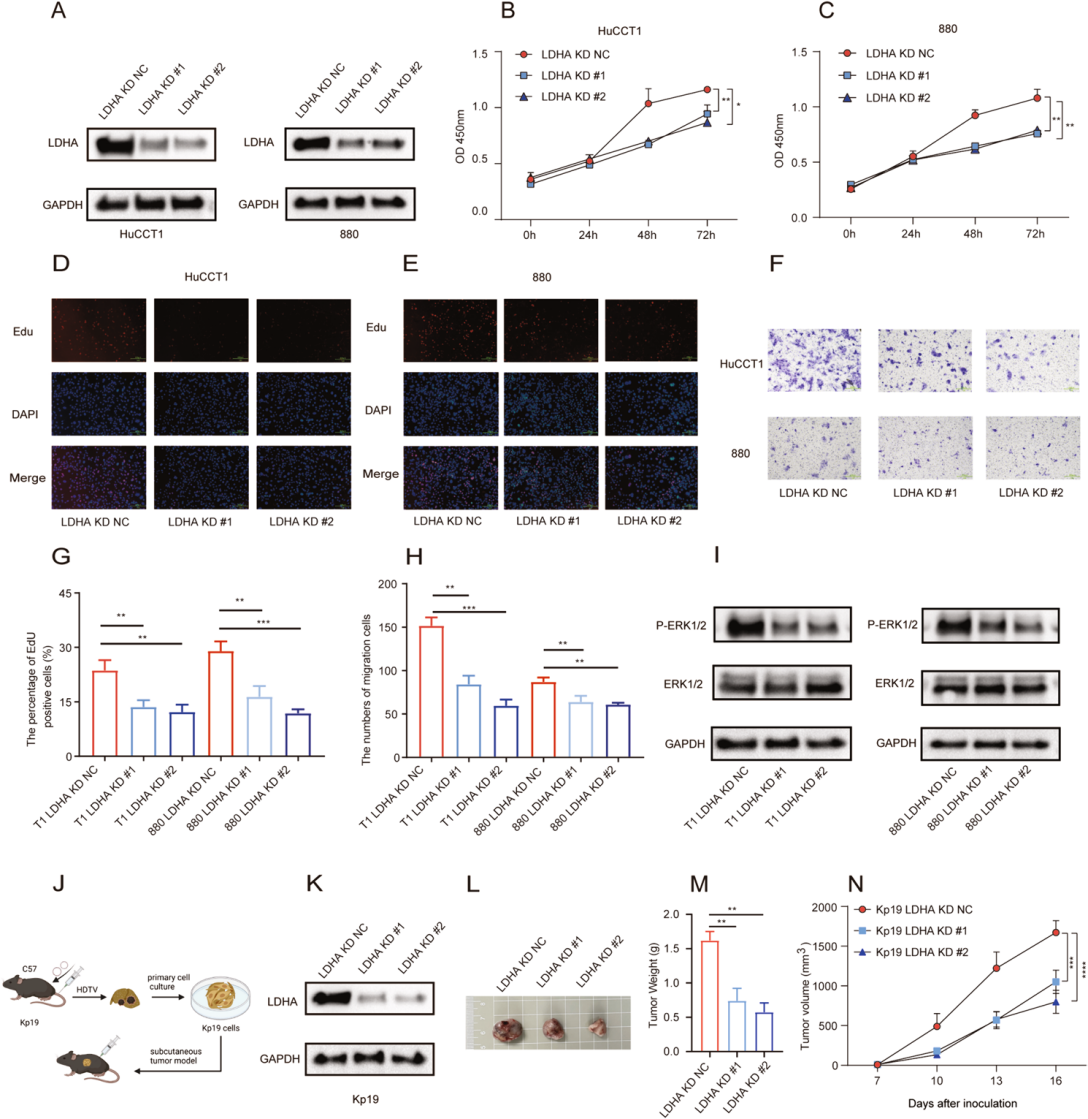
*LDHA* knockdown inhibited tumor growth in vitro and in vivo. The knockdown efficiency of *LDHA* in human iCCA cell lines verified with western blot analysis (**A**). CCK-8 assay results of HuCCT1 and 880 cells transfected with h*LDHA* KD#1, h*LDHA* KD#2, or negative control (NC) (**B** and **C**). EdU assay results of HuCCT1 and 880 cells transfected with h*LDHA* KD#1, h*LDHA* KD#2, or NC (**D**, **E**, and **G**). Transwell assay results of HuCCT1 and 880 cells transfected with h*LDHA* KD#1, h*LDHA* KD#2, or NC (**F** and **H**). Changes in the ERK pathway after *LDHA* knockdown analyzed with western blot analysis (**I**). Flowchart of constructing subcutaneous iCCA model in immunocompetent mice (**J**). The knockdown efficiency of *LDHA* in mouse iCCA cell lines verified with western blot analysis (**K**). Growth of subcutaneous tumors in ICCA mice after LDHA knockdown (**L**, **M**, and **N**). *P < 0.05, **P < 0.01, and ***P < 0.001

As reported, *LDHA* not only is correlated with TIME but also directly regulates tumor growth (Le et al. 2010; Guyon et al. 2022; Jiang et al. 2021). Accordingly, its biological functions in iCCA were further probed. Specifically, HuCCT1 and 880 cell lines with both *KRAS* and *TP53* mutations were chosen to assess the knockdown efficiency of *LDHA* (Fig. 7A). Subsequently, cell proliferation was tested with CCK-8 and EdU assays, and migration was examined with the Transwell assay. The results demonstrated that LDHA knockdown markedly reduced iCCA cell proliferation and migration (Fig. 7B–H). Further, patients were categorized based on their LMRG score and high *LDHA* expression, followed by GSEA. The results unraveled that the mitogen‑activated protein kinase (MAPK) pathway was activated in patients with high LMRG scores (Figure S5A) or *LDHA* expression (Figure S5B). The ERK pathway is one of the most significant pathways in the MAPK pathway. Based on the GSEA results, this study further verified that *LDHA* knockdown inhibited the activation of the ERK pathway (Fig. 7I). With reference to prior studies (Lin, Dai, et al. 2022; Affo et al. 2021), the primary iCCA cell line Kp19 derived from C57BL/6J mice was successfully constructed in this study (Fig. 7J). Subsequent to *LDHA* knockdown (Fig. 7K), Kp19 cells were subcutaneously inoculated into immunocompetent mice. The data confirmed that *LDHA* knockdown substantially repressed tumor growth in mice (Fig. 7L–N). Overall, *LDHA* knockdown may depress tumor growth by blocking the activation of the ERK pathway.

## Discussion

The role of lactate metabolism in tumorigenesis and TIME has been extensively studied in various cancers (Ratter et al. 2018; Thorn et al. 2009; Angelin et al. 2017). However, prior research has predominantly focused on bile acid and lipid metabolism in iCCA (Zhang et al. 2020; Yu et al. 2023, 2020; Martinez et al. 2020; Herraez et al. 2020), and the significance of lactate metabolism in iCCA remains largely undetermined. In this study, 27 prognostic-related genes were identified among 284 LMRGs with the univariate Cox regression model. Moreover, six LMRGs that could serve as potential prognostic biomarkers for iCCA were successfully filtered out with the Boruta algorithm. Based on these six genes, the LMRG score was calculated, which enabled discrimination between iCCA patients with good and poor prognoses. Next, WES data exhibited that *TP53* and *KRAS* mutations were highly enriched in the high LMRG score group. Previous studies have elucidated that increased lactate metabolism was enriched in *TP53-*mutated patients with multiple myeloma (Shah et al. 2018) and *KRAS-*mutated patients with lung adenocarcinoma (McCleland et al. 2013), implicating the potential association of *TP53* and *KRAS* mutations with lactate metabolism in iCCA.

Since TIME is crucial for cancer initiation and progression, this study also analyzed the correlation between LMRG scores and the distribution of immune cells and immune factors such as cytolytic scores, immunoediting scores, and co-inhibitor genes. Our result revealed that immune-activated cells including NK cells were markedly depressed in the high LMRG score group, while immune-suppressive cells such as neutrophils were enriched in the high LMRG score group, which was also validated by correlation analyses and prior research (Deng et al. 2021). Since scRNA-seq is a robust technology to explore the immune microenvironment, scRNA-seq data were obtained from two public datasets and reanalyzed (Ma et al. 2021; Song et al. 2022). The result validated that NK cells were markedly decreased in the high LMRG score group, concordant with prior results that lactate metabolism products were detrimental to the activity of NK cells (Glass et al. 2020; Scott and Cleveland 2016). Due to the limited sensitivity of scRNA-seq to neutrophils, neutrophil variations in scRNA-seq datasets were not analyzed. Given previous findings that neutrophils were enriched in tumors with *KARS* mutations (Lin et al. 2022a; Zhang et al. 2023), neutrophils were included in further experimental verification.

Subsequently, IHC was used for further validation of our findings. Among the variables assessed with the Boruta algorithm, LDHA was the most significant gene associated with the prognosis of iCCA patients (Fig. 1D). LDHA expression was examined, which exhibited a significantly negative correlation between LDHA expression and OS. In addition, CD56 and CD66b expression was also detected since they are widely recognized surface markers of NK cells and neutrophils, respectively. The results demonstrated a positive correlation between LDHA and CD66b expression and a negative correlation between LDHA and CD56 expression. In terms of mechanistic analysis, our study also analyzed pathway alterations in patients with high LMRG scores or *LDHA* expression. The results displayed the significant activation of the MAPK pathway, underscoring the significance of *LDHA* in this signature.

In addition, our study also investigated the impact of *LDHA* on tumor growth with the use of the representative cell lines HuCCT1 and 880. The results demonstrated that *LDHA* knockdown substantially diminished iCCA cell proliferation and migration, similar to conclusions from previous oncology studies (Le et al. 2010; Guyon et al. 2022; Jiang et al. 2021). This result highlights the role of *LDHA* in regulating cell proliferation and migration in iCCA. Regarding mouse experiments, we deemed that the conventional nude mouse xenograft model cannot fully replicate the characteristics of both tumor immunity and tumor metabolism, and iCCA lacks corresponding mouse-derived cell lines. To incorporate key factors of tumor metabolism and the immune microenvironment, a subcutaneous iCCA model was established in immunocompetent mice. The results confirmed the repressive effect of *LDHA* knockdown on tumor growth. Simultaneously, it was found that *LDHA* regulated the ERK pathway within the MAPK pathway. Reportedly, the ERK pathway not only regulates tumor growth but also influences immune cell infiltration in TIME (Yuan et al. 2020; Ullah et al. 2022).

Tumors not only exhaust local energy stores but also generate lactate through anaerobic metabolism, resulting in immunosuppression and tumor growth. Given that glycolysis is largely required for the proliferation of cytotoxic T cells and the production of cytokines, these cells and cytokines are inactive under conditions of low glucose levels and high lactate concentrations (Macintyre et al. 2014). A prior study unveiled that LDHA downregulation partially restored the function of effector T cells, underscoring the significant role of LDHA in mediating the impact of lactate metabolism on these immune cells (Pucino et al. 2019). In addition, another study reported that LDHA knockdown in Pan02 cells (a pancreatic cancer cell line) substantially repressed the tumorigenicity of these cells in mice. Of note, NK cells were revealed to elevate tumor-killing activity after LDHA knockdown, indicating that LDHA may contribute to tumor immune escape by impairing immune cell function (Husain et al. 2013; Brand et al. 2016; Certo et al. 2020). This finding is concordant with our conclusion. Phosphomevalonate kinase 2 (*PMK2*) is another gene implicated in lactate metabolism, which is also essential for the progression of tumors. PKM2 dimerization can translocate into the nucleus to stabilize hypoxia inducible factor 1 subunit alpha (HIF1α) and to induce the expression of glycolytic genes. Previous research elucidated that PKM2 modulated breast cancer cell proliferation through the proteasomal degradation of AU-rich protein tristetraprolin (Huang et al. 2016). Furthermore, PKM2 can also enter mitochondria to phosphorylate BCL2 (an apoptosis regulator), thus facilitating cancer cell adaptation to oxidative stress (Liang et al. 2017). Through single-cell technology, a study systematically analyzed tumor-infiltrating B cells at various stages of colorectal cancer and identified a novel subset of B cells (LARS B) characterized by high expression of leucyl-tRNA synthetase 2 (LARS2). These cells were dispersed within the tumor stroma and their enrichment was positively correlated with the dismal prognosis of patients (Wang et al. 2022). Collectively, these studies on LMRGs underscore the pivotal role of lactate metabolism in tumor progression.

However, our research also has several limitations. Specifically, although our study explored the impact of *LDHA* on tumor cell proliferation and confirmed its relationship with immune cell infiltration in TIME, it remains uncertain whether *LDHA* affects the malignant progression of tumors primarily by influencing the intrinsic growth of tumors or by regulating tumor immunity. In addition, this study failed to extensively elucidate the internal mechanisms through which *LDHA* regulates TIME in iCCA. Hence, further studies on these aspects are warranted.

## Conclusion

In summary, our study developed an LMRG prognostic signature for iCCA patients and obtained the LMRG score for predicting the prognosis of patients. In addition, the high LMRG score group exhibited a high frequency of *TP53* and *KRAS* mutations, high neutrophil cell infiltration, and low NK cell infiltration. *LDHA* knockdown impeded the proliferation and migration of iCCA in vitro and in vivo. These findings offer novel insights into potential targeted treatment for iCCA patients.

### Supplementary Information

Below is the link to the electronic supplementary material.

Supplementary file1 Figure S1. Expression and internal correlations of LMRGs. (A) Expression heat map of the six LMRGs in the cohort of Deng et al. (B) Correlation plot for the six LMRGs in the cohort of Deng et al. (PDF 500 KB)

Supplementary file2 Figure S2. Association of LMRG scores with the prognosis of iCCA. (A) Multivariate Cox regression analysis of LMRG score and TNM stage in the cohort of Deng et al. (B) The correlation between LMRG scores and survival time in the cohort of Deng et al. (PDF 501 KB)

Supplementary file3 Figure S3. A nomogram generated to estimate the survival rate of iCCA patients. (A) The nomogram for predicting the 1- and 3-year OS probabilities in the cohort of Deng et al. (B) Calibration curves of the nomogram in predicting 1- and 3-year OS probabilities in the cohort of Deng et al. (C) ROC curves of the nomogram in the cohort of Deng et al. (D) Decision curves of the nomogram in predicting 1- and 3-year OS in the cohort of Deng et al. (PDF 495 KB)

Supplementary file4 Figure S4. Immune landscape of the high and low LMRG score groups. (A) The boxplot of immune cells in the cohort of Deng et al. (B) The correlation of immune cells with LMRG scores in the cohort of Deng et al. (C) The heat map of cytolytic score-related genes in the FU-iCCA cohort. (D) The correlation of GNLY and GZMM with LMRG scores in the FU-iCCA cohort. * *P* < 0.05, ** *P* < 0.01, and *** *P* < 0.001. (PDF 653 KB)

Supplementary file5 Figure S5. GSEA analysis of iCCA patients with high or low LMGR scores or high or low LDHA expression. GSEA analysis according to LMRG scores (A) and LDHA expression (B) in the FU-iCCA cohort(J). (PDF 1381 KB)

## Data Availability

The original dataset presented in the study are included in the article/Supplementary Material. Further inquiries could be directed to the corresponding author.
