# Supplementary figures and images for: Identification and validation of a lactate metabolism-related six-gene prognostic signature in intrahepatic cholangiocarcinoma
Source: J Cancer Res Clin Oncol. 2024 Apr 16;150(4):199. doi: 10.1007/s00432-024-05723-4 (PMC11021257; doi:10.1007/s00432-024-05723-4)

A

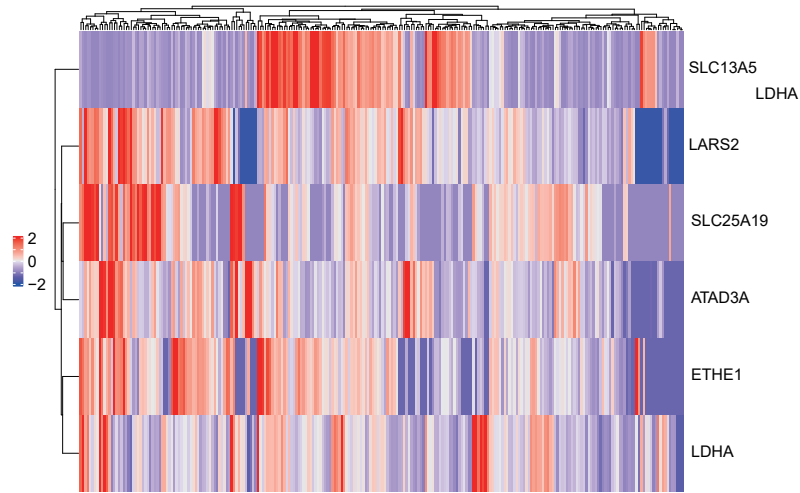

B

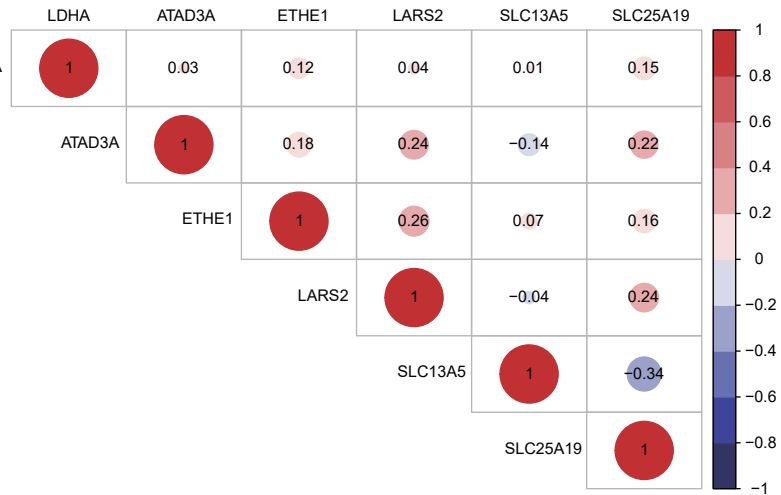

Supplement: Supplementary file 1 — Supplementary file1 Figure S1. Expression and internal correlations of LMRGs. (A) Expression heat map of the six LMRGs in the cohort of Deng et al. (B) Correlation plot for the six LMRGs in the cohort of Deng et al. (PDF 500 KB) [file 432_2024_5723_MOESM1_ESM.pdf]

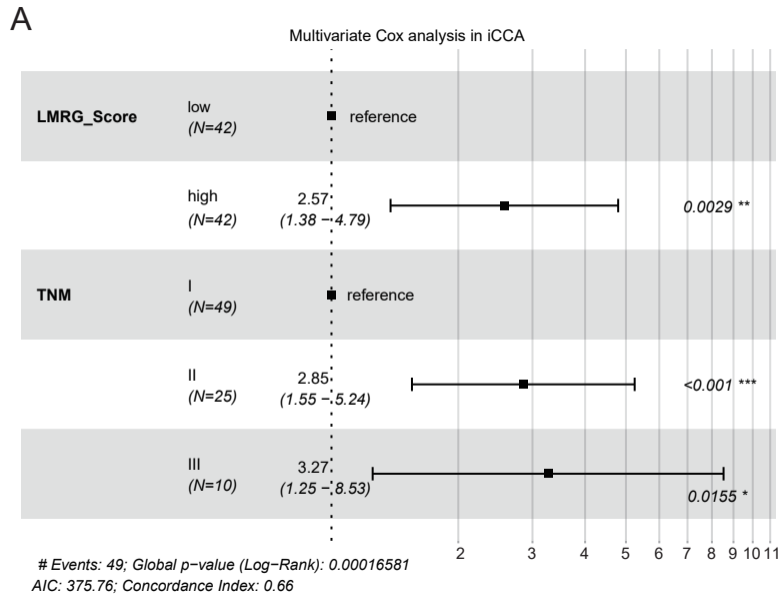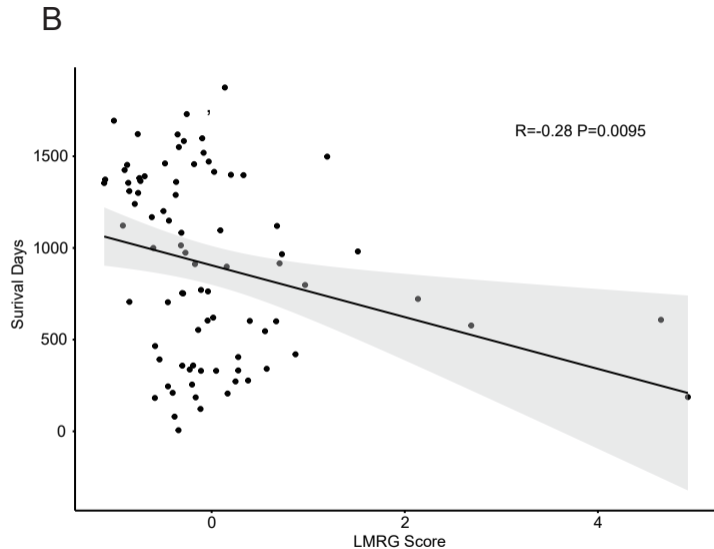

Supplement: Supplementary file 2 — Supplementary file2 Figure S2. Association of LMRG scores with the prognosis of iCCA. (A) Multivariate Cox regression analysis of LMRG score and TNM stage in the cohort of Deng et al. (B) The correlation between LMRG scores and survival time in the cohort of Deng et al. (PDF 501 KB) [file 432_2024_5723_MOESM2_ESM.pdf]

A

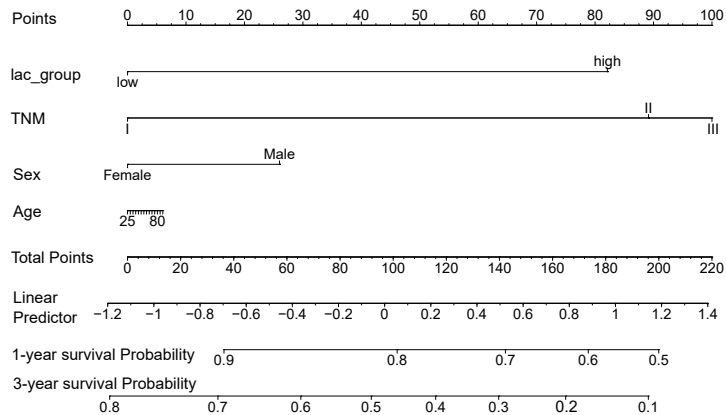

B

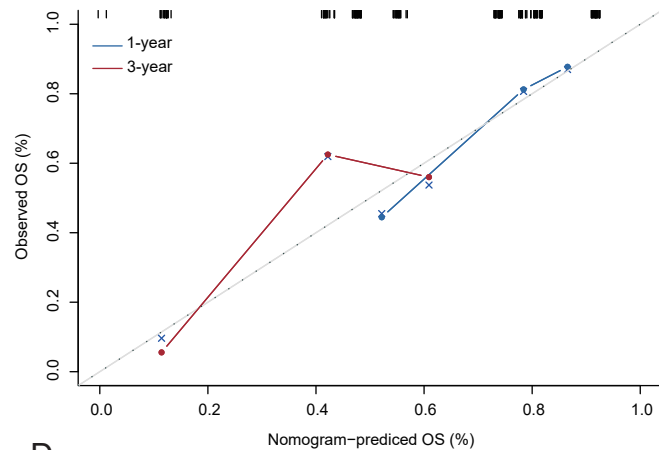

C

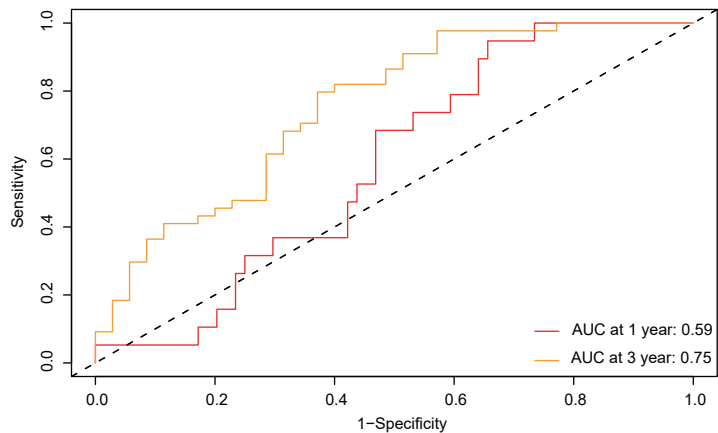

D

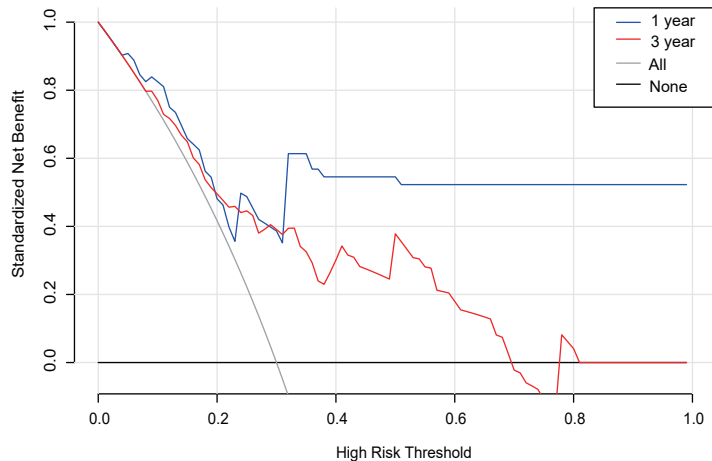

Supplement: Supplementary file 3 — Supplementary file3 Figure S3. A nomogram generated to estimate the survival rate of iCCA patients. (A) The nomogram for predicting the 1- and 3-year OS probabilities in the cohort of Deng et al. (B) Calibration curves of the nomogram in predicting 1- and 3-year OS probabilities in the cohort of Deng et al. (C) ROC curves of the nomogram in the cohort of Deng et al. (D) Decision curves of the nomogram in predicting 1- and 3-year OS in the cohort of Deng et al. (PDF 495 KB) [file 432_2024_5723_MOESM3_ESM.pdf]

A

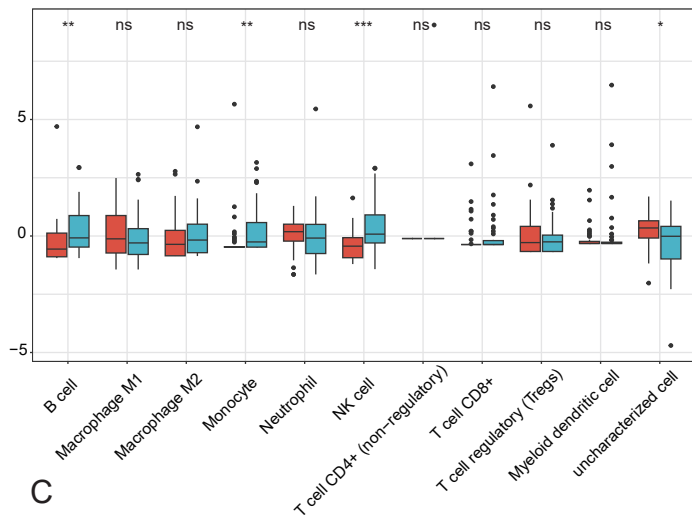

B

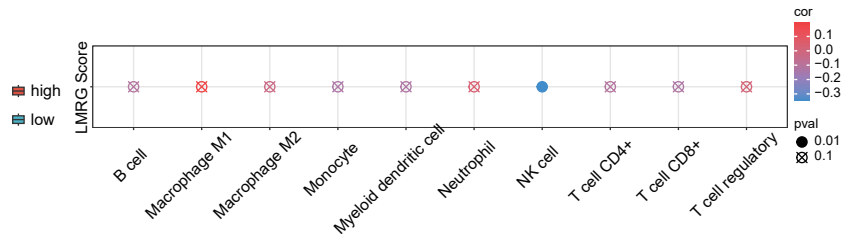

C

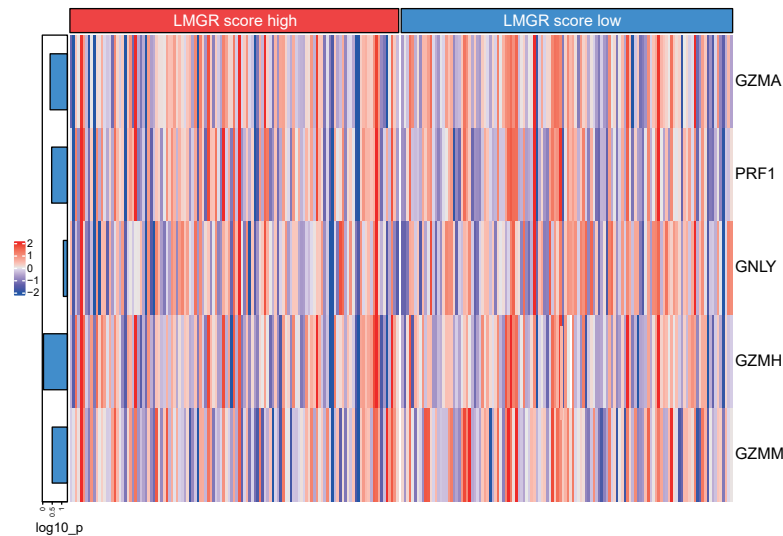

D

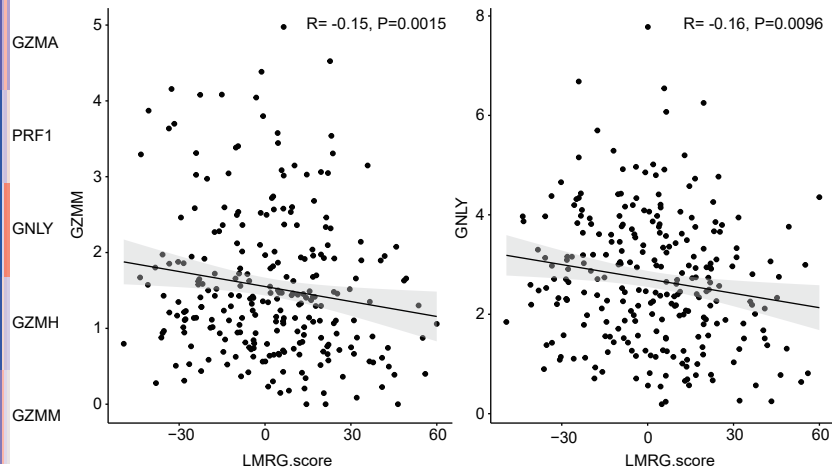

Supplement: Supplementary file 4 — Supplementary file4 Figure S4. Immune landscape of the high and low LMRG score groups. (A) The boxplot of immune cells in the cohort of Deng et al. (B) The correlation of immune cells with LMRG scores in the cohort of Deng et al. (C) The heat map of cytolytic score-related genes in the FU-iCCA cohort. (D) The correlation of GNLY and GZMM with LMRG scores in the FU-iCCA cohort. * P < 0.05, ** P < 0.01, and *** P < 0.001. (PDF 653 KB) [file 432_2024_5723_MOESM4_ESM.pdf]

**A**

GOBP\_MAPK\_CASCADE

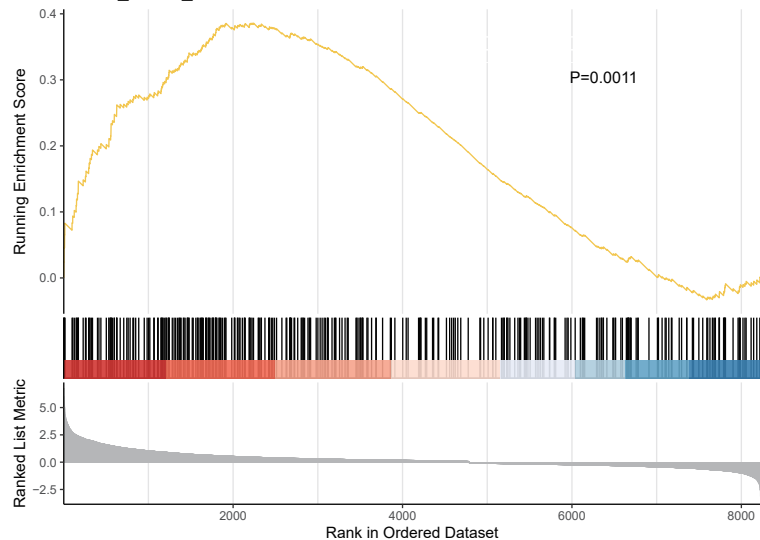**B**

GOBP\_POSITIVE\_REGULATION\_OF\_MAPK\_CASCADE

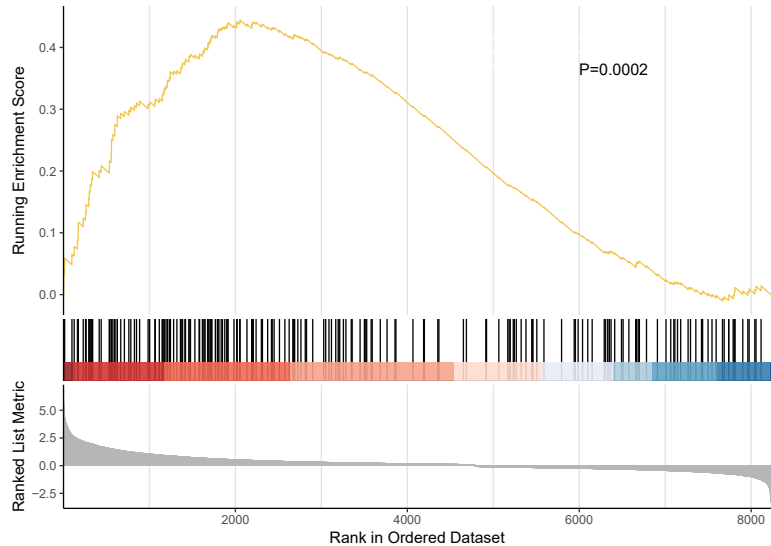

Supplement: Supplementary file 5 — Supplementary file5 Figure S5. GSEA analysis of iCCA patients with high or low LMGR scores or high or low LDHA expression. GSEA analysis according to LMRG scores (A) and LDHA expression (B) in the FU-iCCA cohort(J). (PDF 1381 KB) [file 432_2024_5723_MOESM5_ESM.pdf]
